# From service capacity to spatial equity: Accurately and comprehensively evaluating urban park green space distribution under multi-trips mode

**DOI:** 10.1371/journal.pone.0296629

**Published:** 2024-01-25

**Authors:** Yanhai Zhou, Huili Xie, Xingzhao Liu, Xinke Wang, Zhenfeng Wang

**Affiliations:** 1 College of Landscape Architecture and Art, Fujian Agriculture and Forestry University, Fuzhou, Fujian, China; 2 Innovation Center of Engineering Technology for Monitoring and Restoration of Ecological Fragile Areas in Southeast China, Ministry of Natural Resources, Fuzhou, Fujian, China; King Abdulaziz University, SAUDI ARABIA

## Abstract

The health of city inhabitants is considerably impacted by the urban park green spaces (UPGS). Existing studies lack an assessment of the UPGS equity from different perspectives and are limited to poor accuracy. This study reviews the definition and determination methods of UPGS, as well as UPGS accessibility and spatial equity related studies. Then, a spatial equity evaluation system is established from the dimensions of equity in providing UPGS services and equity in supply and demand matching. This study extrapolates from micro to macro analysis using network big data and census data to pinpoint the population down to the building level in the main urban area of Fuzhou City, China. The network analysis method, improved Gaussian floating catchment area method, Gini coefficient, and Lorenz curve measurement help to evaluate UPGS service euquity, explore the similarities and differences of UPGS fairness under different travel modes. It also helps to understand the service relationship between UPGS service and population demand under objective space, and to pinpoint the weak supply area using the locational entropy method. The results show that: (1) The overall accessibility and per capita accessibility show similar spatial distribution patterns under different travel modes, which are "high around and low in the middle" and "centered on the Minjiang River and dispersed to the north and south, respectively" in study area. (2) The supply of UPGS services in Fuzhou is relatively adequate and at a relatively equity level, while varies in the allocation of UPGS services among sub-districts. The spatial equity of different traveling modes varies widely. (3) The sub-districts on the periphery of the study area should be subject to increased UPGS and improvements to the road network and public infrastructure. Sub-district with high population density and old neighborhoods should implement micro-renewal and build community parks. Our study presents a new idea for spatial equity research.

## 1. Introduction

The rapid expansion of cities has brought about a crisis in the urban environment, resulting in a significant reduction in space for human activities, a decrease in the exchange of activities in some areas [1], and a gradual reduction in the fragmentation of urban space [2]. The Urban Park Green Space (UPGS) was supposed to bring benefits to the city, increase tourism and economic investment, and create a healthy, high quality of life for urban residents [3]. However, its function of providing communication and a sense of community has been negatively impacted in China, and it is seen only as a remnant space of rapid urban expansion. Previous studies have shown that UPGSs are recreational infrastructures for residents of high-density urban areas, are meant to meet their daily public activities [2,4], effectively reducing the chances of suffering from being overweight and obese [5], cardiovascular disease [6], and mental health issues [7]. Incorporating green infrastructure at the community scale can yield significant environmental benefits, such as improving air quality [8], reducing noise [9], and maintaining biodiversity [10]. The importance of UPGS in the enhancement of quality of life cannot be overstated. Therefore, it is important to focus on equity issues.

The "spatial equity" of urban green space planning is roughly divided into three stages: regional equality, spatial balance, and social equality [11,12]. The core purpose of regional equality is to solve the problem of the availability of parkland and to ensure that the amount of parkland services per capita in different regions is as equal as possible. At this stage of urban planning, the general orientation of functional zoning and efficiency was not considered, and human needs and spatial layout were not considered, resulting in an uneven distribution of parkland and separation of people and greenery. Spatial balance gradually emphasizes service efficiency and begins to focus on specific zones and quantities of facility allocation. However, at this stage, the allocation of UPGS only focuses on equal amounts, without considering socio-spatial differentiation and social group differentiation, and it pays insufficient attention to spatial layout rationality, which leads to an imbalance in the supply and demand for park green spaces in local lots and the waste of land resources. However, due to the differences in regional development and the development goals of "people-oriented" and "matching supply and demand" in the new era, the criteria for judging the equity of UPGS should be given newer meanings. Currently, research on the equity of UPGS is still in the exploratory stage. To enhance the precision of the study, the service supply side was differentiated according to the characteristics of UPGS [13], and the rationality of the spatial layout was comprehensively explored [14]. At this stage, most studies have introduced methods from different disciplines, such as overall social equity performance indicators from economics [15,16] and locational entropy [17,18]; geographically weighted regression analysis from geography [19], Moran’s index [20], and spatial autocorrelation analysis [21], which greatly enriched the spatial equity analysis, however a unified framework for spatial equity in urban public green spaces has not yet been proposed, and the applicability of research methods has yet to be explored (Table 1).

**Table 1:** The development of spatial equity.

Developmental stage	Target	Typical indicators	Existing problem
**Regional equality**	Make sure there are UPGS in the city	Tokyo, Japan: Park green area per capita 4.6m^2^	UPGS distribution is uneven and UPGS is separated with people
		USA: National Recreation and Parks Association proposed the Park Law	
		China: Green area per capita, Proportion of green space in the park	
**Spatial balance**	Ensure that equal amounts are allocated to UPGS between regions	Sheffield, England: The minimum size of UPGS within 0.3, 2.5, and 10 kilometers around residential buildings was set	There are problems of supply and demand imbalance and waste of land resources
		China: The minimum scale and per capita area of UPGS within the living circle of 5, 10 and 15 min are stipulated	
**Social equality**	People-oriented planning to achieve supply and demand matching	Scholars from various countries are exploring, but a unified evaluation system has not been formed yet.	There are top-down allocations led by the government and lack direct public participation in contribution

Existing UPGSs are often planned only in terms of location, ignoring the relationship between the characteristics of UPGS services and spatial distribution of demand. With the development of modern cities, there should be more innovative concepts and methods for evaluating urban park services, requiring not only that parks meet "quantitative" standards, but also that they provide better services under the concept of equal opportunity. By grasping the data dividend of the network era, integrating multi-source big data into the application practice of urban research, and perceiving the physical and social space of the city from a new dimension, the accuracy of the research will also be greatly improved. Therefore, this study proposes an accurate equity evaluation systems for UPGS. Four new concepts are proposed to enhance evaluation significance. 1) The selection of the three most common modes of travel for urban residents, namely walking, cycling, and driving, to explore the similarities and differences in the actual services of UPGSs under different modes of travel. 2) Considering the objective variability of UPGS, differential measurements are made according to the actual utility to ensure that the uniqueness of UPGSs and the complementarity of the system work together, while the fuzzy set method is used to deal with the distance impedance coefficient and setting of the UPGS service range. 3) Evaluating the spatial equity of UPGSs based on the dual dimensions of actual service capacity and population accessibility to clearly understand the spatial variability of park service supply and the spatial match between UPGS services supply and population demand. 4) The concepts of overall accessibility and per capita accessibility are introduced. The population data, which are accurate to the level of building units, are summed up to the sub-district scale for evaluation, and the differences between overall and per capita characteristics are explored from microscopic extrapolation to macroscopic analysis.

This study starts from different travel modes, characterizes the population quantity of building monoliths as the demand quantity, differentiates the service effectiveness on the service supply side according to the nature of different UPGS, and jointly explores the rationality of UPGS layout under different travel modes using both service supply and supply–demand matching dimensions. We also discusses the applicability of the research method in order to provide different ideas for the study of spatial equity of UPGS and provide scientific support for the optimization of urban green space systems and site-specific policies.

## 2. Materials and methods

### 2.1. Study area

Fuzhou is located at 25°15′–26°39′N and 118°08′–120°31′E. It is a prefecture-level city and the capital of Fujian Province, with a total area of 11,968 km2 and a subtropical monsoon climate. By the end of 2020, the total resident population of Fuzhou was 8,291,000, and the regional GDP was 100,202,000,000 Yuan; the urbanization rate rose from 62.0% in 2010 to 72.5% in 2020, and urban population gathering will cause tension in urban resource constraints and a shortage of public resources. Research on the scientific evaluation of the status of existing urban park services and the guidance for the equitable supply of urban green space through spatial planning methods to reduce the spatial mismatch and spatial waste between green space resources and population demand caused by unreasonable development are yet to be improved.

**Fig 1:**
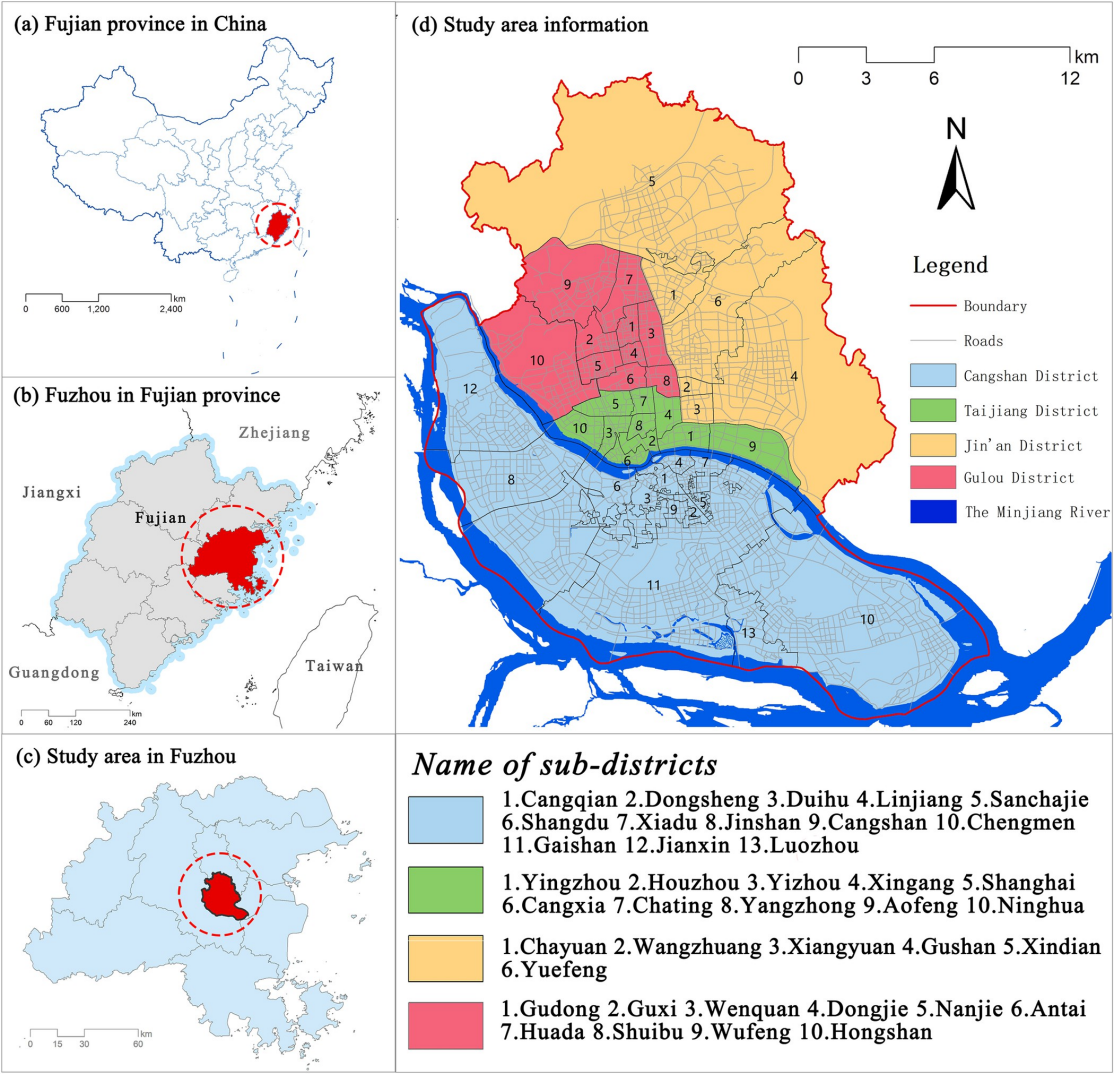
Location of the districts and sub-districts in the city center of Fuzhou.

The study area is the main urban area of Fuzhou City, including four districts of the Cangshan District, Jinan District, Gulou District, and Taijiang District (part of the builtup area). By the end of 2021, the total area of the main urban area of Fuzhou City is approximately 311.8 km^2^ (Fig 1).

### 2.2. Data source and preprocessing

#### 2.2.1. Urban park green space (UPGS)

**Fig 2:**
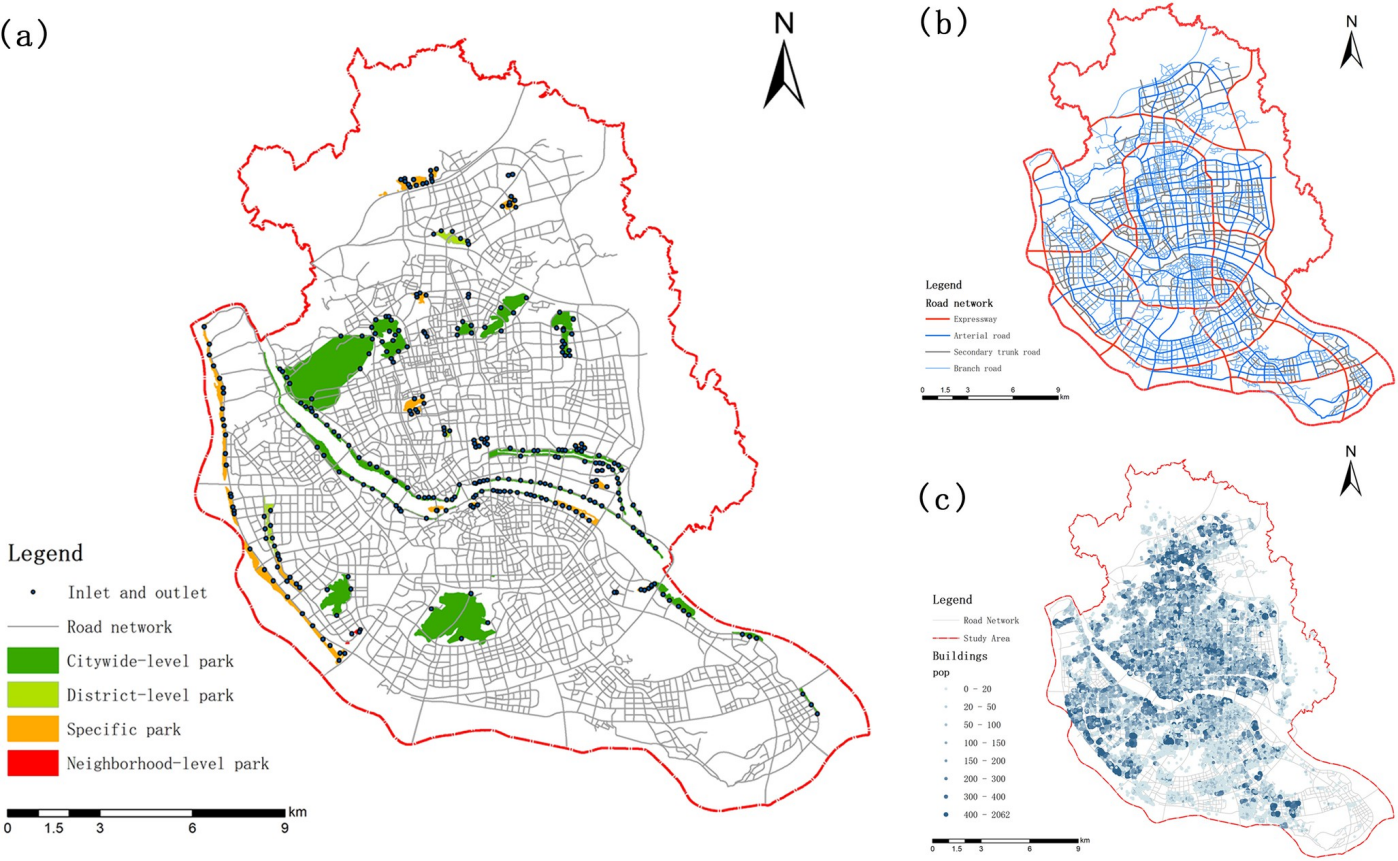
Basic research data. (a) Urban parks distribution; (b) Road network classification; (c) Population distribution map.

The Fuzhou City Park Management Measures, published by the Fuzhou Landscape Bureau in 2019 based on the national standard, define a city park as "a place with a good gardening environment and better service facilities, with the functions of touring and resting, ecology, beautification, disaster prevention and avoidance, etc., and open to the public." [22] The Fuzhou Green Space System Plan evaluates and defines UPGS based on factors such as size, function, and site specifics, and categorizes urban parks into citywide comprehensive parks, regional comprehensive parks, community parks, special category parks, community parks, and sub-district green spaces. Due to the limited area of sub-district green space and the inadequate facilities equipped, a total of 43 parks were selected with reference to previous literature (Fig 2).

In this study, based on the park information directory recorded by the Fuzhou City Landscape Bureau, The Fuzhou City Green Space System Plan (2016–2020) [23] was compared with High-resolution remote sensing image maps and field surveys for calibration and adjustment to form ArcGIS recognizable surface vector data. Four types of citywide comprehensive parks, regional comprehensive parks, community parks, and specialized parks were finally selected, and the actual entrances and exits of the parks were marked with poi data and field surveys to establish a database of UPGSs.

By the end of 2021, 43 UPGSs had been built in the main city of Fuzhou, with a total area of 129.5 km^2^. Seventeen of these are citywide comprehensive parks, three regional comprehensive parks, five community parks, and 18 special category parks. People have different willingness to spend time in different types of parks, and the better the parks are, the more time and energy people tend to spend on visitation [13]. In this study, the travel time thresholds for city-level parks, regional-level parks, community-level parks, and specific parks were set at 40, 30, 10, and 15 minutes, respectively, with reference to previous studies [15] (Table 2).

**Table 2:** All levels of built-park information in the Fuzhou main area (by the end of 2021).

Type	Count (ratio)	Area/hm2 (Ratio)	Travel time threshold
**City-level park** **Reginal-level park** **Community-level park**	17 (39.5)	1173.7 (81.8)	40min
	5 (11.6)	5.2 (0.4)	20min
	3 (7.05)	48.2 (3.4)	10min
**Specific park**	18 (41.9)	208.1 (14.4)	15min
**Total**	43 (100)	1435.1 (100)	

#### 2.2.2. Road network

The Open Map Street platform was used to obtain and extract open-source road network data in and around the study area. We combined Google Maps high-resolution images and the Fuzhou City Comprehensive Traffic Plan (2010–2020) for data correction, where roads were defined as four attributes: expressway, trunk road, secondary road, and branch road, and assigned speed values of 60 km/h, 45 km/h, 30 km/h, and 20 km/h, respectively [24,25] (Fig 2).

#### 2.2.3. Population

To obtain more accurate results, this study used building units as the population calculation unit and crawled the building outline and building height from Baidu Map. Using ENVI 5.1 software, we used the remote sensing information extraction method and obtained the residential land vector data by geometric correction, alignment, false color synthesis, stitching and cropping of high-resolution remote sensing images, and visual interpretation and human-computer interaction interpretation with reference to the existing LUCC classification system.

The building outline and height were crawled from Baidu map, and residential land space was overlaid to filter out residential buildings. Simultaneously, the building height was divided by the average building height per floor to obtain the number of building floors that were entered into the attribute table. The population of each building was estimated based on the population data of each sub-district from the 7th National Census, published by the Fuzhou City Bureau of Statistics (Fig 2.). The calculation is as follows.


$$D_{k}=\frac{\left(RA_{K}\times n\right)}{RA}\times SP,$$ (1)

$$D_{k}=\frac{\left(RA_{K}\times n\right)}{RA}\times SP,$$ (2)

Where *D*_*k*_ is the population of the *k*th building; *RA*_*k*_ is the contour area of that building and *n* is the number of building floors; *RA*_*k*_ is the total residential area of the Sub-district where building *K* is located; *SP* is the total population of that Sub-district; *i* = 1,2…K; 30 buildings were randomly selected as samples, and the number of households in these buildings was surveyed by following the average household size of 3.5 people (The seventh census data of Fuzhou), the calculation results of the above equation were tested, and the average accuracy rate was at 82%, so it is considered that this estimation can better reflect the population of residential areas. All data sources and acquisition times are shown in (Table 3).

**Table 3:** Data source.

Data set	Data sources
Urban park green space	The Fuzhou City Green Space System Plan (2016–2020) (http://ylj.fuzhou.gov.cn/, accessed on 21 October 2019) High-resolution remote sensing images (https://www.gditu.net/, accessed on 6 June 2021)
Population	Census data (http://tjj.fuzhou.gov.cn/zz/fztjnj/, accessed on 10 November 2021) High-resolution remote sensing images (https://www.gditu.net/, accessed on 6 June 2021) Baidu maps (https://map.baidu.com/, accessed on 8 June 2021)
Road network	Open Map Street (www.openstreetmap.org, accessed on 4 February 2022)
Administrative boundaries	Resource and Environmental Science and Data Center of Chinese Academy of Sciences (https://www.resdc.cn/, accessed on 10 November 2021)

### 2.3. Methodology

**Fig 3:**
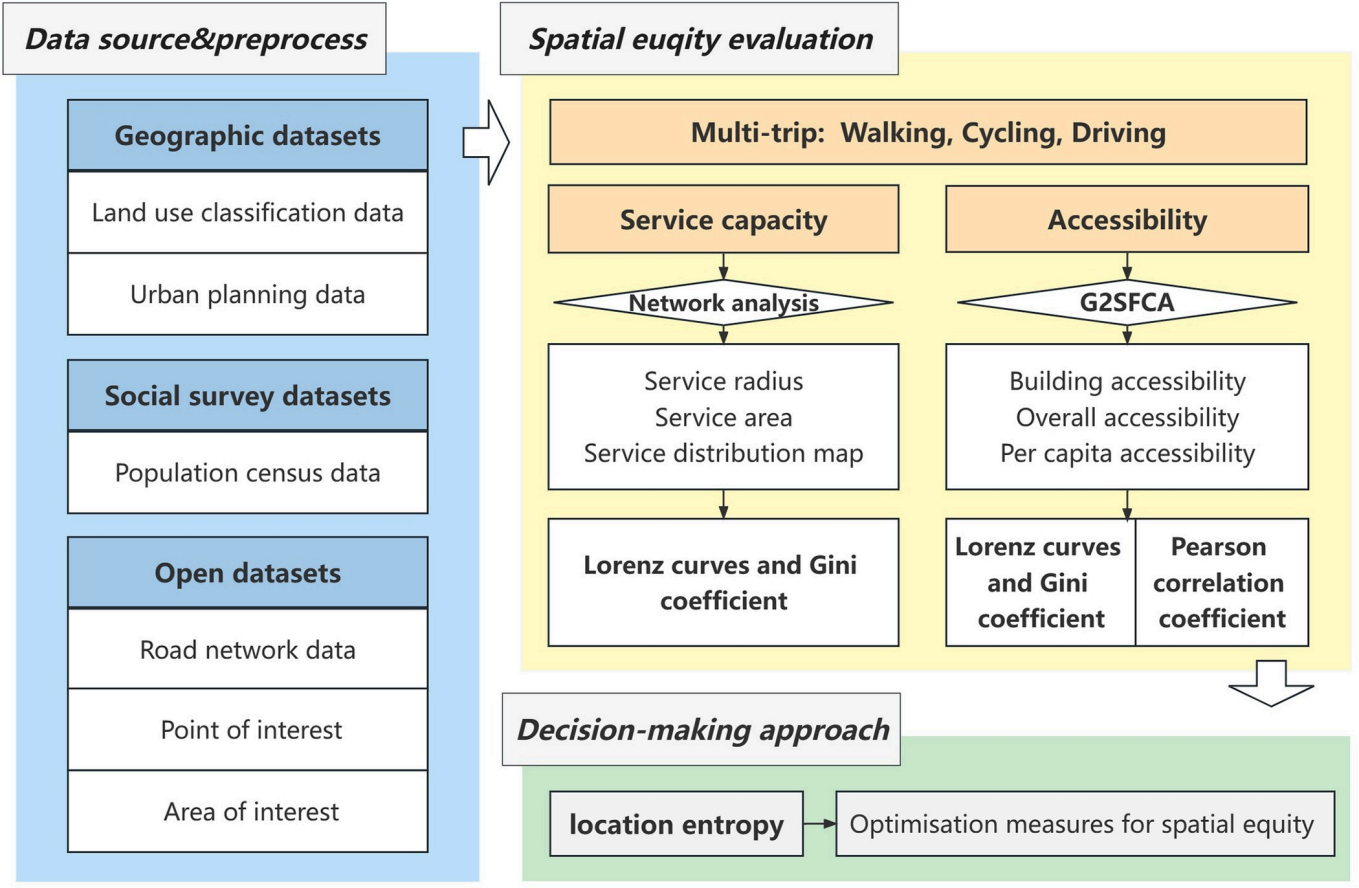
Methodological framework.

We propose a methodological framework for evaluating and improving the spatial equity of UPGS based on multi-source big data and government public documents. This equity evaluation system starts from two dimensions: service supply equity and supply–demand matching equity, which quantitatively analyzes the spatial variability of UPGS service supply and population accessibility under different travel modes. Then, we introduces the Gini coefficient, Lorenz curve, and Pearson correlation analysis to evaluate the degree of equity under the dual dimensions, and finally locates the inequitable areas with the locational entropy method and proposes corresponding improvement measures to provide scientific decision support for urban planning and UPGS layout (Fig 3).

#### 2.3.1. Effective service level of UPGS

According to the willingness of residents to travel to different urban parks, the travel times for city-level park, district-level park, neighborhood-level park and special parks were set as 40 min, 20 min, 10 min, and 15 min, respectively, and the service coverage ratio of each type of park was calculated using a network analysis method according to different travel modes. Drawing on the research method of Tang et al. [26], the service level of UPGS was calculated as the ratio of the sum of the effective service area of UPGS in a spatial unit to the area of the spatial unit in which it is located, using the formula:

$$LD=\frac{M_{j}}{A_{j}},$$ (3)

Where *LD* is the service level of UPGS in spatial unit j, *M*_*j*_ is the sum of the effective service area of green space in spatial unit *j* (i.e., the total amount of UPGS resources in spatial unit j), and *A*_*j*_ is the area of spatial unit *j*.

#### 2.3.2. Accessibility measurement model of UPGS

Most accessibility applications measure the spatial attributes of public facilities in cities and are the basis for spatial equity evaluation. Hansen et al. [27] first introduced the concept of accessibility as an opportunity for interactions between nodes in a transportation network. As an important metric for public service fairness evaluation, accessibility has been widely used and improved in academic circles [28], mainly through spatial analysis, network analysis, and the two-step floating catchment area method. The spatial analysis is simple to calculation and easy to operate, and the buffer zone [29,30] and the nearest method [31,32] were included. However, It ignores obstacles such as actual road networks, and data accuracy problems, such as the determination of the center point and service radius, so the measurement results often differ significantly from the real situation. The network analysis method is based on realistic urban road networks for simulation calculations, which can realistically simulate the actual capacity of roads [33,34]. This study also used this approach to study the service provision capacity of UPGS. Compared with other accessibility analysis methods, the two-step floating catchment area method considers the interaction relationship between supply and demand and derives a series of improved algorithms according to different situations [35,36]. For example, Enhanced-2SFCA [37], KD2SFCA [38], Huff-2SFCA [35] and Gaussian-2SFCA [39]. However, these researches are based on the medium and macroscopic scale and are not sufficiently detailed, thus representing a significant gap with the actual situation. There is less unused space in high-density cities, and the refined management mode can improve all aspects of urban planning, providing high-quality and efficient public services, and realizing the sustainable development of cities. Taking precision evaluation as the first guide and considering its applicability, this study adopts the improved Gaussian two-step moving search method as the accessibility evaluation method, which is calculated from a microscopic perspective accurate to the level of building units.

(1) Improved-G2SFCA

The innovation of this study is that the actual entrance or exit of the city park was used as the starting point of the calculation and the network analysis method was used to replace the Euclidean distance with the actual distance of the road network, which greatly increases the reliability of the calculation results and is more suitable for the actual situation. In addition, owing to the differences in UPGS types, areas, and service capacities, the accuracy of the accessibility evaluation results will be affected if the same search threshold radius is used. Therefore, this study selected the three preferred travel modes of Fuzhou, namely walking, cycling and driving, to comprehensively considered the search threshold of different types of park. With reference to previous studies, the average speeds of walking and cycling were selected as 5 km/h and 12 km/h, respectively [18], and the travel by car was set at 60 km/h for expressways, 45 km/h for main roads, 30 km/h for secondary roads, and 20 km/h for branch roads. The travel speed and willingness time were multiplied to obtain the search radius by comprehensive estimation (Table 4).

**Table 4:** Search threshold (d0).

Type	Walking	Cycling	Driving
**City-level park** **Reginal-level park** **Community-level park**	3000 m	8000 m	20000 m
	1600 m	4000 m	10000 m
	800 m	2000 m	5000 m
**Specific park**	1200 m	3000 m	7500 m

The improved two-step mobile search method was divided into two steps [40]. In the first step, the actual entrance or exit point data of the park are used as the starting point, and the building units are the destination points for analysis. The buildings *k* in the search space range *d*_*0*_ of urban park *j* were screened, the number of all populations in *d*_*0*_ were aggregated, weights were assigned according to the distance decay law using a Gaussian function, and these weighted populations are summed and aggregated to calculate the supply or demand ratio *R*_*j*_:

$$R_{j}=\frac{S_{j}}{\sum_{k\in \{d_{jk}⩽d_{0}\}}G(d_{jk})D_{k}},$$ (4)

Where *D*_*k*_ is the population number of each building *k*, *d*_*kj*_ is the actual road network distance between locations *k* and *j*. For parks with multiple entrances, the road network distance from the residents to the nearest entrance is selected while building *k* is in the search domain (i.e., *d*_*kj*_ ≤ *d*_*0*_); *S*_*j*_ is the area of urban green space *j*; *G(d*_*kj*_*)* is the Gaussian decay function considering the spatial friction problem, and its expression is:

$$R_{j}=\frac{S_{j}}{\sum_{k\in \{d_{jk}⩽d_{0}\}}G(d_{jk})D_{k}}(djk<d0),$$ (5)

In the second step, the analysis is performed with the building as the starting point and the park green space data as the destination point. All urban parks *j* in the search domain *d*_*0*_ are found separately, and the supply demand ratio *R*_*j*_ of these UPGSs is summed up on the basis of the Gaussian decay function to obtain the spatial accessibility *A*_*k*_ of building *k* based on the distance cost, whose higher value indicates a higher degree of accessibility [39].


$$A_{k}=\sum_{j\in \{d_{kj}⩽d_{0}\}}\;G(d_{kj})R_{j},$$ (6)

The results of park accessibility calculated by the two-step moving search method have the meaning of green space per capita in a broad sense; therefore, they can be compared with urban green space per capita as a basis for evaluating accessibility, especially for conducting cross-sectional comparisons of different units in the region.

(2) Overall accessibility and per capita accessibility

The distribution of the building units was too scattered to be considered a complete evaluation unit. Therefore, the overall accessibility and per capita accessibility of a Sub-district are introduced to evaluate accessibility at the sub-district scale by inverting from micro to macro. The overall accessibility *AJ* of sub-district *i* is the sum of the accessibility of all buildings in the Sub-district and the product of the building population, and the per capita accessibility *AP* is the ratio of the overall accessibility to the population of the sub-district.

$${AJ}_{i}=\sum \;(A_{ik}\times D_{ik}),$$ (7)

$${AP}_{i}=\frac{{AJ}_{i}}{{SP}_{i}},$$ (8)

where *D*_*ik*_ is the population of building *k* in Sub-district *i*, *A*_*ik*_ is the accessibility calculation result of building k, and *SP*_*i*_ is the total population of sub-district *i*. The overall and per capita accessibility results were visualized to evaluate the spatial distribution pattern of accessibility under different travel modes.

#### 2.3.3. Spatial equity evaluation based on Gini coefficient and Lorenz curve

The Lorenz curve method is often used to explore the fairness of national income in national distribution. Recently, the Lorenz curve has been widely used in the fields of urban public resource allocation and the spatial layout of green spaces to reflect the equity level. The Gini coefficient has values between 0 and 1. The smaller the Gini coefficient, the more reasonable the level of the spatial distribution of resources among urban residents, and the fairer its spatial distribution (Table 5). In this study, the Lorenz curve was introduced to analyze the fairness level of the spatial distribution of green space in the main urban area of Fuzhou. The calculation formula is as follows.


$$GINI=1-\sum_{i=1}^{n}(q_{i}+q_{i-1})(p_{i}-p_{i-1}),$$ (9)

Because the Lorenz curve and Gini coefficient of this study differ in the calculation methods of different evaluation dimensions, only one of them was chosen here as a representative. where *GINI* is the Gini coefficient, *q*_*i*_ is the cumulative percentage of the Sub-district service area (accessibility), and *p*_*i*_ is the cumulative percentage of sub-districts (buildings).

**Table 5:** Gini coefficient and equity relation reference.

**GINI**	<0.2	0.2–0.3	0.3–0.4	0.4–0.6	>0.6
**Degree**	Completely equal	More equal	Relatively reasonable	Great discrepancy	Wide disparity

#### 2.3.4. Spatial inequity identification based on locational entropy method

The location entropy of UPGS was calculated using the Sub-district as the spatial unit. The locational entropy of UPGS service capacity of each Sub-district is the ratio between the effective service area share of UPGS in the Sub-district and the service area share in the whole study area, while the accessbility location entropy is the ratio between the effective service area enjoyed by the population per capita in the Sub-district and the public green space resources enjoyed by the population per capita in the whole study area. The equations are as follows.

$${LQ}_{i}=\frac{\left(\frac{T_{i}}{Pi}\right)}{\frac{T}{P}},$$ (10)

where *LQi* is the location entropy of the service capacity (accessbility) of UPGS in Sub-district *i*, *Ti* is the service area (accessible population) of UPGS in Sub-district *i*, *Pi* is the area (population) of Sub-district *i*, *T* is the service area (total accessible population) of UPGSs in the main urban area of Fuzhou, and *P* is the area (total population) within the main urban area of Fuzhou.

#### 2.3.5. Pearson correlation coefficient

The Pearson correlation coefficient was mainly used to measure the degree of linear correlation between the two variables. We use the Pearson correlation analysis in the dimension of accessibility to explore the relationship between population, area, and accessibility and to judge whether the spatial distribution of UPGS is equal in a comprehensive way. Simultaneously, the correlation degree between different accessibility measurement methods and different travel modes is explored to enrich the connotation of spatial equity research. Suppose there exist two sets of data of the same length, *X* = {x1, x2,…, x_n_}, *Y* = {y1, y2,…, y_n_}; then, the expressions are:

$$p=\frac{cov(X,Y)}{\sigma_{X}\sigma_{Y}},$$ (11)

where *cov (X*,*Y)* denotes the covariance of *X* and *Y* and *σ*_*X*_ and *σ*_*Y*_ are the standard deviations of the data. The Pearson’s correlation coefficient was between -1 and 1. When the linear correlation between two variables is enhanced, the correlation coefficient tends to 1 or -1. When *Y* increases with the increase in *X*, the correlation coefficient is greater than 0, and there is a positive correlation; when *Y* decreases with an increase in *X*, the correlation coefficient is less than 0, and the closer it is to -1, the more significant the negative correlation; when the correlation coefficient tends to 0, there is no correlation between the two variables [41].

## 3. Results

### 3.1. Evaluating UPGS spatial equity based on service supply

#### 3.1.1. Effective service supply of UPGS

**Fig 4:**
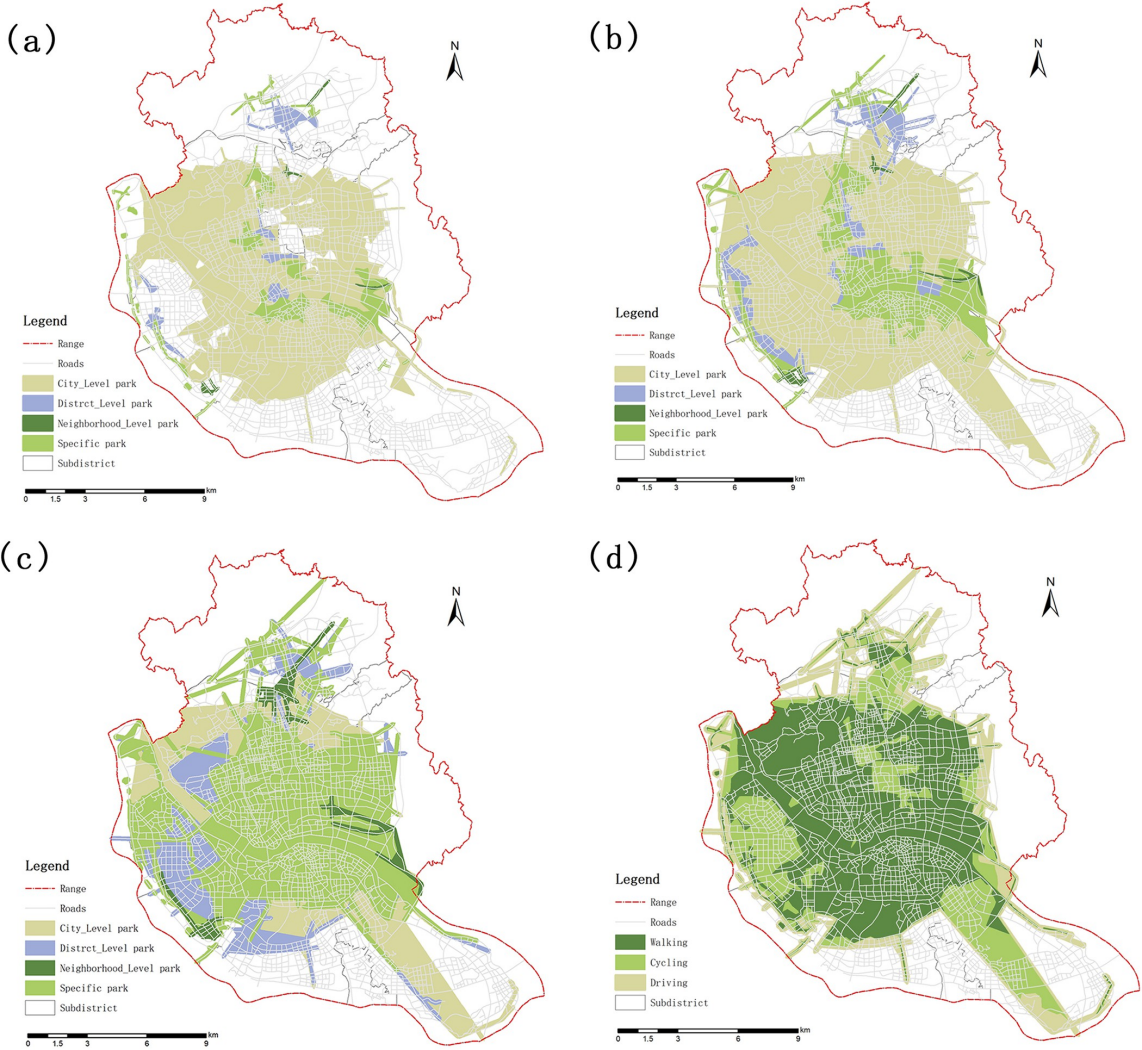
Spatial distribution of UPGS service capacity. (a)Walking; (b) Cycling; (c) Driving; (d) All kinds of transport modes.

**Fig 5:**
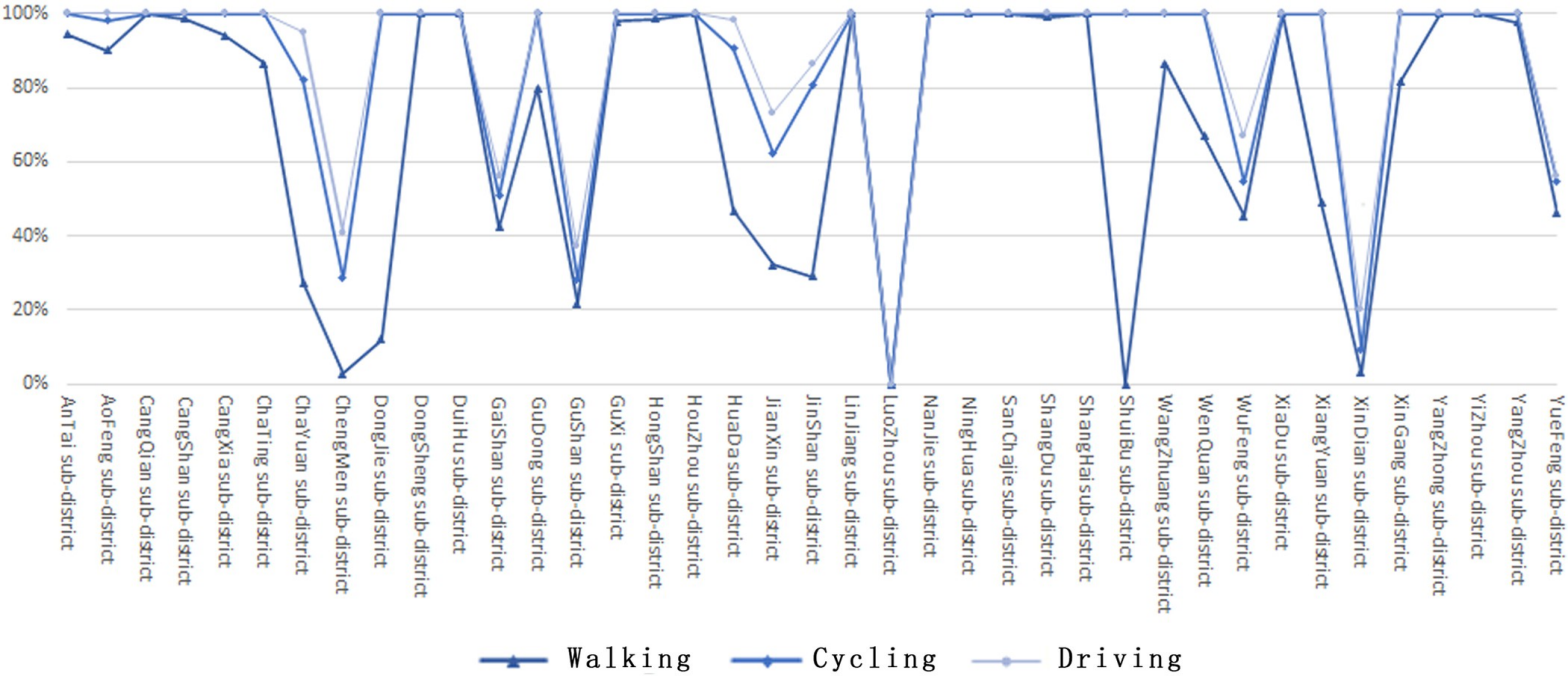
The proportion of effective service area of each sub-district.

The effective service area of the UPGS under the three travel modes are superimposed, showing a spatial distribution pattern of "spreading outward from the center" (Fig 4), with the locations at the edge of the study area are not covered by UPGS services. The percentage of UPGS service area varies widely by travel mode. In the walking case, there are 25 Sub-districts with more than 50% coverage of UPGS, less than 20% are Chengmen, Dongjie, Luozhou, Shuibu, and Xindian Sub-districts, and the effective service area of UPGS in Shuibu and Luozhou sub-districts in the walking case is 0. In the cycling case, there were 11 Sub-districts with full coverage of UPGS service, reaching 28.2% of the total. There are two Sub-districts with less than 20% of the service area, namely the Luozhou and the Xindian sub-districts. In the driving situation, the overall Sub-district coverage ratio is high for all sub-districts, with 64.1% of the Sub-districts achieving 100% coverage, only the sub-districts of Luozhou and Xindian are at a lower level. Overall, with the upgrade in travel mode, the effective service area of parks in the study area increased (Fig 5), with service coverage in the walking, cycling, and driving scenarios accounting for 33.4%, 48.5%, and 56.0% of the study area, respectively. Dongsheng, Duihu, Nanjie, Sanchajie, and Yangzhong sub-districts performed the best, with full UPGS service coverage in all three travel scenarios. On the other hand, Luzhou Sub-district did not enjoy park services in any of scenarios.

#### 3.1.2. Spatial equity analysis based on service supply

**Fig 6:**
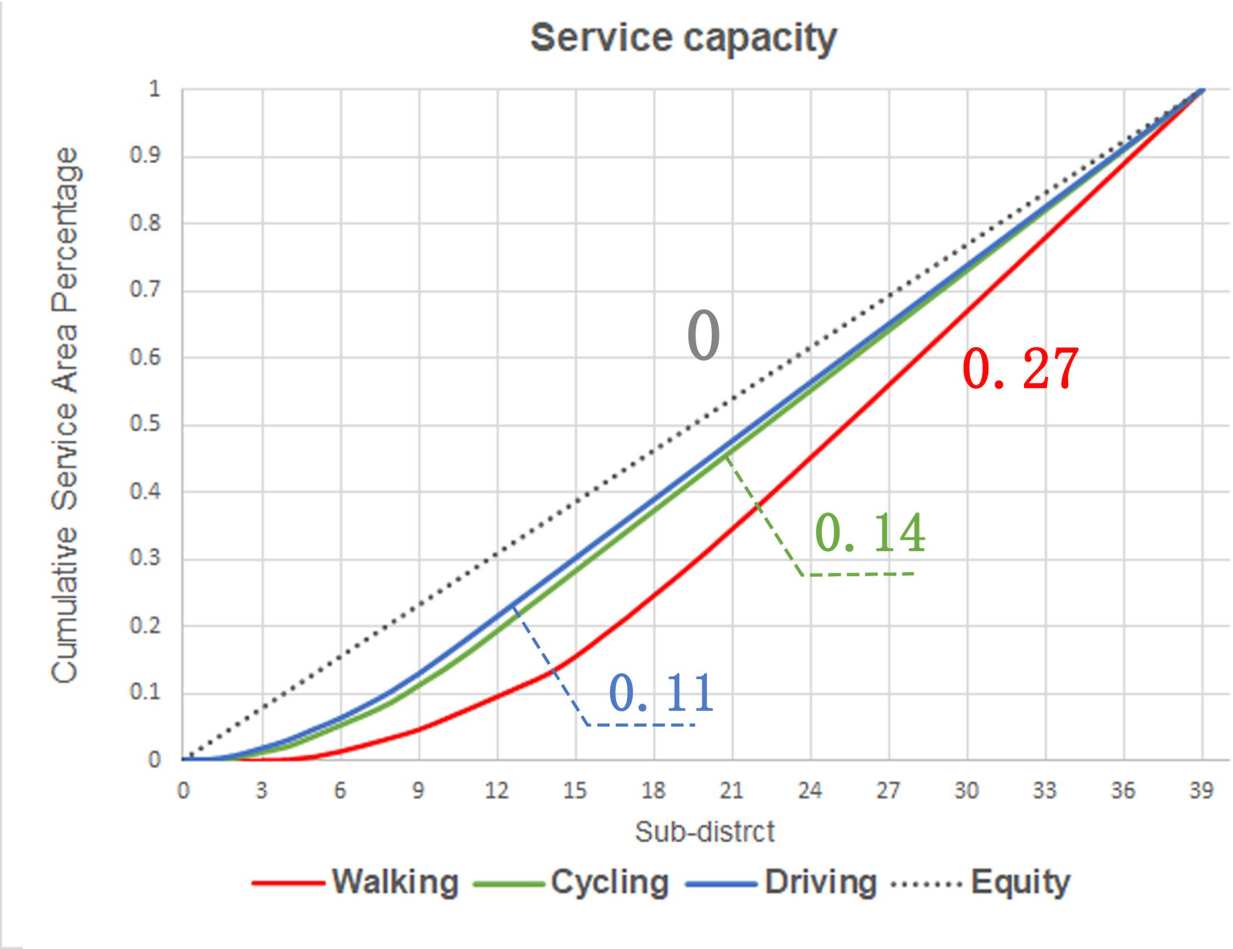
Lorentz curve of service capacity in Fuzhou main urban area.

The Lorenz curve graph was drawn from the calculation results of the effective service area of each UPGS to judge the fairness of the spatial allocation of UPGS in the service supply dimension (Fig 6). The numerical magnitude of the Gini coefficient reflects the degree of difference in the allocation of urban park resources between Sub-districts under different traffic modes. The Lorenz curve indicates the allocation of park green space resources in different Sub-districts under different travel modes, and a line with a slope of 1 is regarded as the line of perfect equality. The further away from the absolute fairness line, the greater the degree of difference in service supply between Sub-districts and vice versa.

The Gini coefficient of UPGS service capacity is 0.27 when choosing the walking travel mode, which is at a relatively fair level. The Gini coefficients of service capacity in the case of cycling and driving are 0.14 and 0.11, respectively, which are extremely fair. Therefore, the main city of Fuzhou has a good supply of UPGS services, the sub-districts can enjoy UGPS services more equally among themselves, and the spatial allocation is quite equitable.

#### 3.1.3. Locational entropy analysis based on service supply

**Fig 7:**
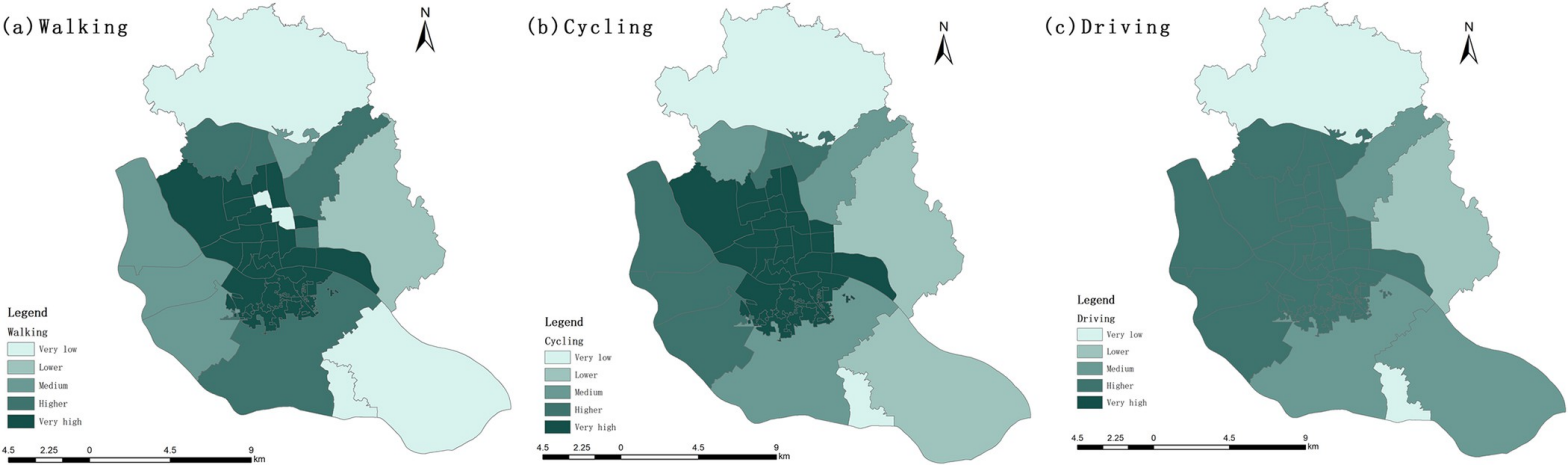
Spatial distribution of service capacity location entropy.

To identify the weak areas of UPGS service supply and provide scientific support for urban planning, this study introduces the locational entropy method. If the locational entropy of a Sub-district spatial unit is greater than 1, it indicates that the UPGS service supply of the Sub-district is higher than the overall level of the study area. On the contrary, it indicates that it is lower than the overall level of the study area. Based on previous studies, the sub-district locational entropy was divided into five levels with 0.5, 0.75, 1.2, and 2 as the separation values, and the area with low locational entropy was analyzed as the focus of the study (Fig 7).

Overall, the overall UPGS supply in the study area is good, with the central area enjoying a higher supply than the peripheral sub-districts, and the Xindian, Gushan, Chengmen, and Luozhou Sub-districts are at a lower level. Different travel modes show similar spatial distribution patterns. However, only when the travel mode is walking, the Dongjie and Shuibu Sub-districts are in areas with low values of locational entropy. In summary, the sub-districts which are located at the periphery of the study area, have a weak supply of UPGS services, and planning and construction should be conducted for these areas first.

### 3.2. Evaluating UPGS spatial equity based on accessibility

#### 3.2.1. Accessibility analysis

**(1) Accessibility based quantitative population analysis**

Based on the G2SFCA analysis, the accessibility of buildings in the study area was obtained, and then graded using the natural breakpoint method to obtain five levels of building accessibility grading: very high, high, medium, low, and very low. The number and percentage of the population graded for different travel modes of accessibility were obtained by summing the population of buildings at each level and comparing it with the total population of the study area (Table 6). With the upgrade of travel mode, the number of people with higher and very high accessibility increased from 8.56% to 34.17%, while the number of people with very low accessibility showed a decreasing trend from 74.75% to 53.18%. The population with very low accessibility when the travel mode was driving accounted for 45.47%.

**Table 6:** Accessibility grading of urban park under different transport modes.

Transport modes	Accessibility grading	Population	Proportion/ (%)
**Walking**	Very high (1.34–3.25)	94795	3.17
	Higher (0.53–1.34)	131279	4.39
	Medium (0.28–0.53)	190460	6.36
	Lower (0.10–0.28)	338919	11.33
	Very low (0.00–0.10)	2236849	74.75
**Cycling**	Very high (0.55–1.94)	137689	4.60
	Higher (0.27–0.55)	331251	11.07
	Medium (0.13–0.27)	432490	14.45
	Lower (0.04–0.13)	499651	16.70
	Very low (0.00–0.04)	1591221	53.18
**Driving**	Very high (0.43–5.27)	140214	4.69
	Higher (0.30–0.43)	439783	14.70
	Medium (0.22–0.30)	582736	19.47
	Lower (0.06–0.22)	468903	15.67
	Very low (0.00–0.06)	1360666	45.47

**(2) Accessibility based spatial layout analysis**

**Fig 8:**
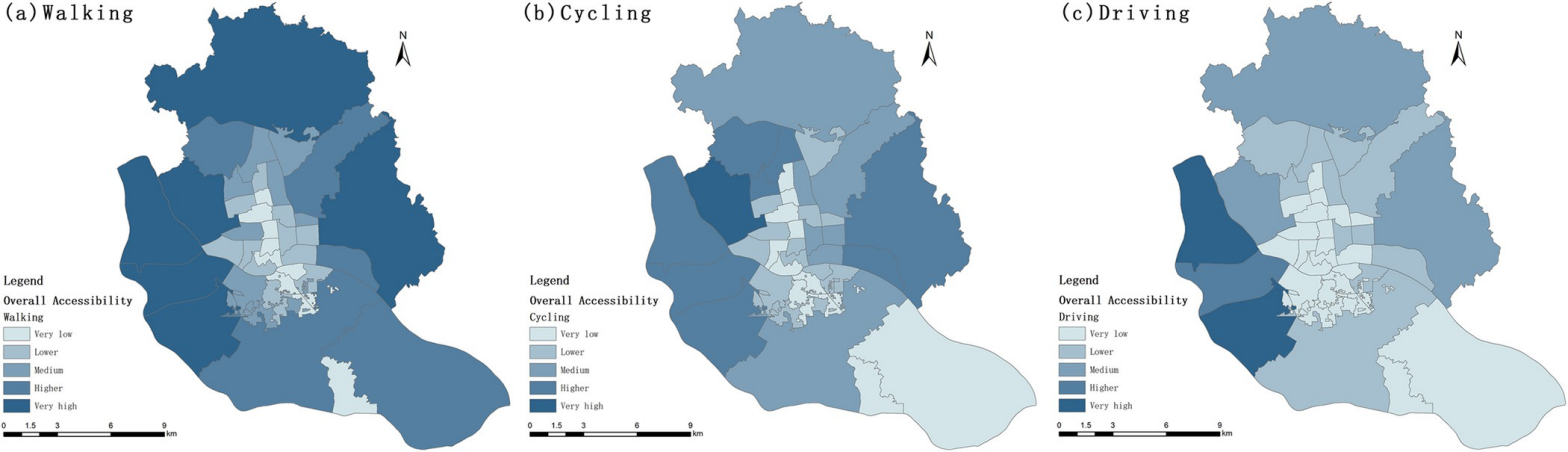
Spatial distribution of urban park overall accessibility.

The overall accessibility of the sub-districts showed a spatial distribution pattern of "high around and low in the middle"(Fig 8). The small sub-districts in the middle of the study area and the southeast side of the Luozhou Sub-district have the lowest accessibility, while the larger sub-districts on the northwest side generally have higher accessibility. The overall accessibility ranking of the Dongsheng and Xiadu sub-districts continues to rise with the upgrading of travel modes, while the ranking of the Cangxia and Shang hai sub-districts continues to fall. The Aofeng sub-district, Gushan sub-district, Hongshan sub-district, Jianxin sub-district, and Jinshan sub-district under the three travel modes are ranked high while Antai Sub-district, Cangqian Sub-district, Dongjie Sub-district, Luzhou Sub-district, and Sanjiajie Sub-district are at a lower level.

**Fig 9:**
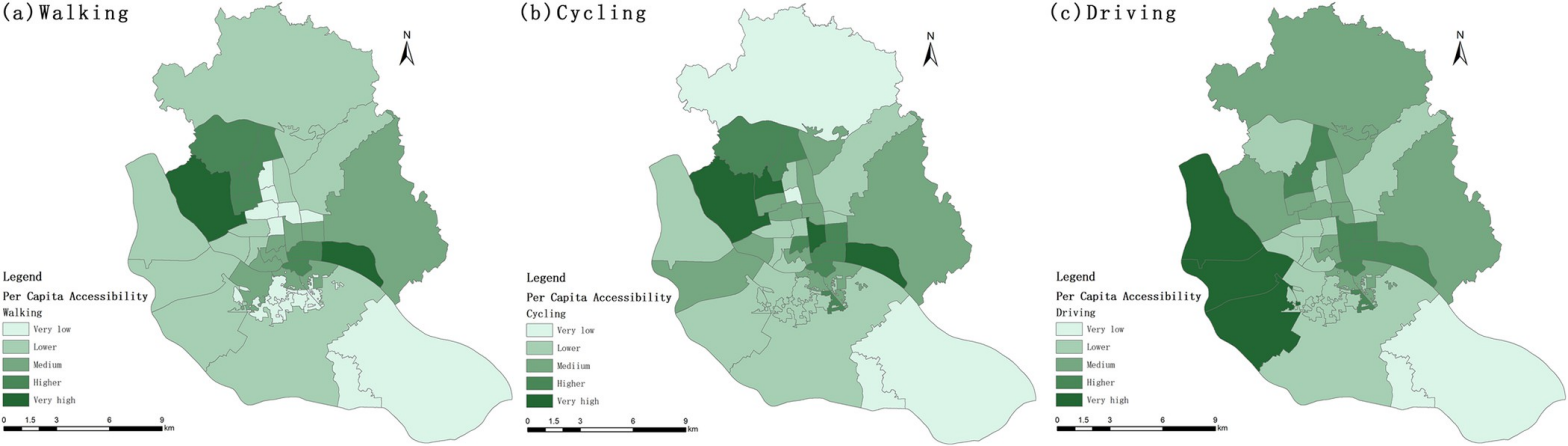
Spatial distribution of urban park Per capita accessibility.

The per capita accessibility is generally higher in the sub-districts located near the riverside parks on both sides of the Minjiang River, showing a spatial distribution pattern of "Minjiang River as the center, dispersing to the north and south, respectively(Fig 9). With the upgrading of travel mode, the accessibility per capita of each sub-district is on the whole on the rise, only the accessibility of Luozhou Sub-district and Chengmen Sub-district in the southeast of the study area and only Luzhou and Chengmen sub-districts, which are located in the southeast of the study area, have been maintained at a very low level. With the upgrade of travel modes, the ranking of Antai and Dongsheng sub-districts continue to rise, while the ranking of Wufeng, Aofeng, and the six group sub-districts (Cangxia, Houzhou, Ninghua, Shanghai, Yizhou, and Yingzhou Sub-districts) located in the central part of the study area continue to decline. The Aofeng, Guxi, and Yingzhou sub-districtd ranked better under the three travel modes, while Chengmen, Dongjie, and Luozhou sub-districts had low rankings.

With the upgrading of travel modes, the overall accessibility changes more slowly than the per capita accessibility ranking, and the spatial distribution pattern of overall accessibility and per capita accessibility varies greatly. The top three sub-districts in terms of per capita accessibility under walking conditions were Aofeng, Hongshan, and Yingzhou sub-districts, while Jianxin, Hongshan, Xindian, Gushan, and Jinshan sub-districts ranked high in terms of overall accessibility and much higher than per capita accessibility. In contrast, the Aofeng, Linjiang, Nanjie, and Yingzhou sub-districts have better per capita accessibility and worse overall accessibility. This spatial heterogeneity was also found in the accessibility results for both cycling and driving modes of travel.

#### 3.2.2. Spatial equity analysis based on accessibility

**Fig 10:**
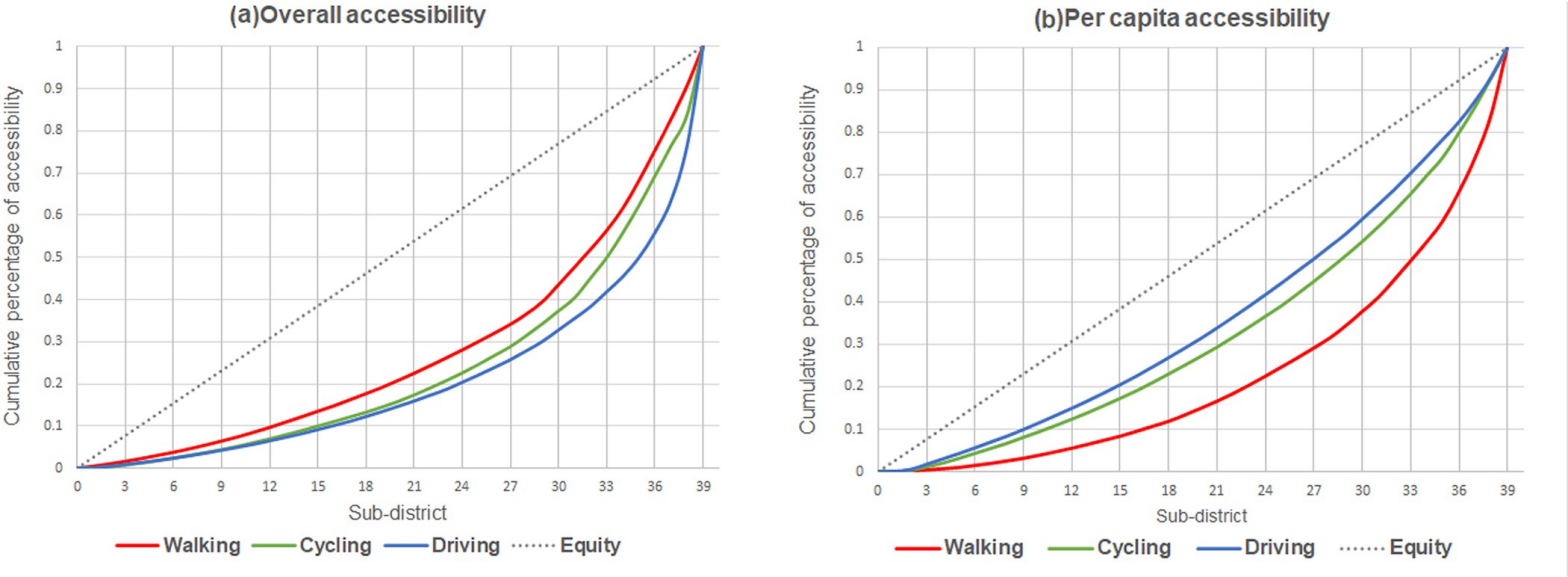
Lorentz curve of accessibility in Fuzhou main urban area.

The Lorenz curve was plotted based on the results of the accessibility calculation to measure the fairness of UPGS allocation in the population accessibility dimension (Fig 10).

The overall accessibility of UPGS varies widely between sub-districts, and the spatial variability becomes more obvious as the Gini coefficient increases with the escalation of travel modes (Table 7). Combined with the results of the Pearson’s correlation analysis, the correlations between population and the overall accessibility of sub-districts of walking, cycling, and driving were 0.977, 0.620, and 0.813, respectively (Table 8). The distribution of UPGS resources and the distribution of the sub-district population all showed a strong positive correlation. This indicates that, from the perspective of the overall accessibility of sub-districts, the arrangement of UPGS matches the population demand, and the distribution of resources is relatively reasonable.

**Table 7:** The results of Gini coefficient.

Type	Walking	Cycling	Driving
Building accessibility Overall accessibility Per capita accessibility	0.88	0.79	0.68
	0.45	0.53	0.58
	0.54	0.35	0.28

**Table 8:** Correlation analysis results for accessibility.

Type	W_O	W_P	C_O	C_P	D_O	D_P	POP	Area	O
W_O W_P C_O	1								
	0.230	1							
	0.764	0.621	1						
C_P	0.115	0.832	0.623	1					
D_O	0.786	0.054	0.525	0.044	1				
D_P	0.335	0.310	0.390	0.523	0.688	1			
POP	0.977	0.043	0.620	0.056	0.813	0.314	1		
Area	0.763	-0.114	0.278	-0.256	0.475	0.000	0.829	1	
P	--	--	--	--	--	--	--	--	0.203

* W: Walking; C: Cycling; D: Driving; P: Per capita accessibility; O: Overall accessibility.

The difference in per capita accessibility between sub-districts is relatively small. The Gini coefficient of per capita accessibility of sub-districts in walking mode is 0.54, showing strong inequity. The Gini coefficient was 0.35 when cycling, and the distribution of UPGS was relatively reasonable. The driving mode has the weakest polarization phenomenon compared to the first two modes and is in a more equitable state. The correlations between population and per capita accessibility under the three travel modes were 0.043, 0.056, and 0.314, respectively, which are basically uncorrelated.

#### 3.2.3. Locational entropy analysis based on accessibility

In order to evaluate the "spatial matching" relationship between the distribution of UPGS resources and population distribution, we use the locational entropy method.

There were 21, 21, and 19 sub-districts with locational entropy greater than 1 for the three travel cases, that is, 53.8%, 53.8%, and 48.7% of the sub-districts had a per capita green space area higher than the average level of the study area, respectively (S1 File). The level of per capita enjoyment of UPGS resources also changes under different travel modes: with the escalation of travel modes, the location entropy values of the Jinshan Sub-district, Xiangyuan Sub-district, and Xindian Sub-district show an increasing trend; for Duihu Sub-district, Hongshan Sub-district, Nanjie Sub-district, Shangdu Sub-district, and Yuefeng Sub-district, the location entropy decreases, while the Wangzhuang Sub-district rises and then decreases.

**Fig 11:**
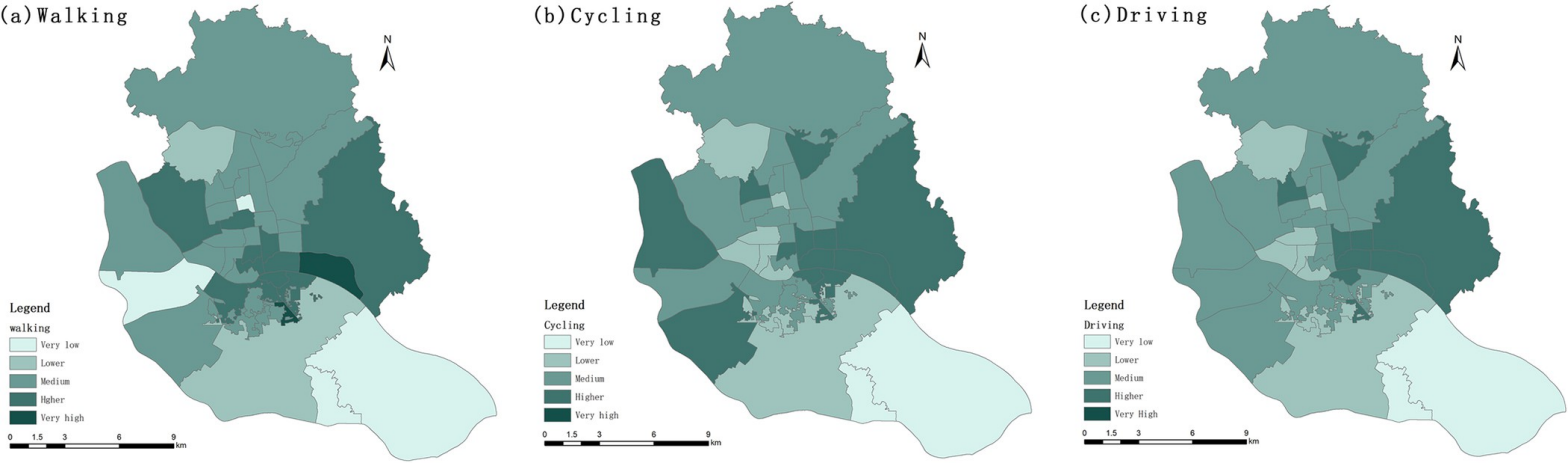
Spatial distribution of accessibility location entropy.

In general, the per capita area of green space in the northwestern part of the main urban area of Fuzhou is higher than that in the southeastern part of the main urban area (Fig 11); Yingzhou Sub-district, Linjiang Sub-district, Aofeng Sub-district, Xingang Sub-district and Gushan Sub-district in the central part of the main urban ar-ea have a higher level of park resources per capita; Chengmen Sub-district and Luozhou Sub-district in the southeast, and Wufeng Sub-district and Dongjie Sub-district in the northwest are at a lower level. In terms of the specific spatial distribution pattern of different travel modes, when the travel mode is walking, the high-level sub-districts are concentrated on both sides of the Minjiang River and gradually dispersed outward; the urban park services in Dongjie Sub-district, Jinshan Sub-district, Chengmen Sub-district, and Luzhou Sub-district are much smaller than the average. The high-level sub-districts in the cycling case are more dispersed and distributed in the western, central, and eastern parts of the study area, and the low value areas are also interspersed. The overall spatial distribution is more balanced in the case of driving, with the highest values in the five sub-district groups in the central-eastern part of the study area, and the lowest values in the sub-districts of Luozhou and Chengmen. In summary, residents of the Luozhou Sub-district, Chengmen Sub-district, Dongjie Sub-district, Wufeng Sub-district, and four sub-districts along the Minjiang River (Ninghua Sub-district, Yizhou Sub-district, Cangxia Sub-district, and Shanghai Sub-district) cannot enjoy better UPGS services. They should be built as the first area for urban planning.

### 3.3. Similarities and differences of spatial equity evaluation in dual dimensions

Spatial equity analysis under both dimensions shows that the UPGS in the main city of Fuzhou is better constructed, and the spatial allocation is more equitable. The Gini coefficient results show that the equity of the service supply dimension is higher than that of the supply–demand matching dimension, and the equity of service supply, architectural accessibility, and per capita accessibility of UPGS increases with the improvement of travel mode.

The location entropy results showed spatial variability under different dimensions. The low-level zones under the service supply dimension are distributed in the periphery of the study area, with the Xindian, Gushan, Chengmen, and Luozhou sub-districts being typical service undersupply zones. The low-level zones under the supply–demand matching dimension are scattered throughout the study area, with the Luozhou, Chengmen, Dongjie, Wufeng, and the four group sub-districts along the Minjiang River showing weak service configurations under all three travel modes. The Luzhou and Chengmen sub-districts are low in both dimensions and are the primary areas of concern for urban planning and construction, and increasing the construction of UPGS should be the primary improvement measures. The Xindian and Gushan sub-districts, also located at the periphery of the study area, perform better in matching supply and demand despite the lack of service supply. It indicates that these two sub-districts have a smaller population distribution due to environmental or social reasons, and the smaller service supply just meets the population. The four group sub-districts along the Minjiang River have a better level of service supply, but due to the large population and high service demand, the supply is less than the demand, and the actual UPGS services that the population can enjoy are insufficient. In summary, urban construction should be developed under a comprehensive analysis and evaluation of both the service supply and supply demand matching dimensions for planning decisions.

## 4. Discussion

### 4.1. A new system for evaluating the equity of UPGS

This study establishes a new evaluation system for the spatial equity of UPGSs, taking different modes of travel as the entry point and evaluating UPGSs in two dimensions. Their advantage over previous studies is their precision and comprehensiveness. To maximize the accuracy of the research results, we use the actual entrance or exit as the starting point for the calculation of the service supply side and differentiate according to the characteristics of UPGS, using the network data and the results of the seventh national census to pinpoint the population demand to the level of building units.

Improvements were made separately under the two-dimensional evaluations. Previous studies on park service capacity remain in the service area to sub-district area ratio[24], this study analyzes the service area to sub-district area ratio and population reachable UPGS area ratio, and introduces the district entropy method to compare the service variability of spatial units horizontally. It not only evaluates the service level of suburban UPGS but also locates the weak supply, providing a scientific basis for accurate planning of urban construction. The difference from the previous accessibility evaluation is that this study introduces the concepts of sub-district accessibility per capita and overall accessibility, and we evaluate the accessibility from the summation of the accessibility precisely to the level of building units to the sub-district scale, from microscopic extrapolation to macroscopic analysis. It provides a new idea for accessibility evaluation and fairness evaluation based on the accessibility.

From the culculation results, the per capita accessibility and overall accessibility of some sub-districts, such as the Aofeng and Xingang sub-districts, are both at a high level, while both accessibility of Luozhou and Dongjie sub-districts are at a low level. However, more sub-districts show the divergence phenomenon of high overall accessibility-low per capita accessibility or low overall accessibility-high per capita accessibility. This spatial divergence phenomenon occurs in all three modes of travel. We combined with Table 7 the correlation between overall accessibility and per capita accessibility for the three modes of travel is 0.23, 0.62, and 0.68, respectively, with less correlation in the case of walking and significant positive correlation in the case of cycling and driving. The population has a significant positive correlation with overall accessibility and almost no correlation with per capita accessibility, indicating that the higher the population of a sub-district, the higher the overall accessibility. The arrangement of UPGS matches the population demand, and the distribution of resources is relatively reasonable [25], while per capita accessibility is not related to the population size. Therefore, when evaluating the accessibility of parkland at the sub-district scale, the per capita accessibility has more reference value, while correlating the overall accessibility of the sub-district with the population. The results can, to a certain extent, discern the rationality of resource distribution.

The spatial equity research framework of this study makes three contributions. First, it comprehensively evaluates the equity under the dual dimensions of UPGS services and population accessibility. Second, improvements were made from data sources to the service supply, demand, and travel modes to ensure the accuracy of the study. Third, the applicability and relevance of the evaluation were explored based on scientific calculations to make a reference for future studies.

### 4.2. Similarities and differences in spatial equity under different travel modes

The spatial equity of UPGSs under the three travel modes of walking, cycling, and driving varies widely, consistent with previous studies’ results [16]. From walking to biking to traveling by car, the effective service area of UPGS within the study area increased, the number of sub-districts with very low locational entropy values decreased, the per capita accessibility of each sub-district showed an overall upward trend, the inaccessible population gradually decreased, and the spatial distribution became more equitable. In other words, if residents are willing to travel at the same time, to a certain extent, choosing transportation modes that can travel longer distances will allow UPGS services to benefit more space, and more people will enjoy quality UPGS services. The per capita UPGS area will increase accordingly, and the spatial distribution of UPGS services will be more equitable.

Under different travel modes, the locational entropy of UPGS service supply does not change significantly, showing an overall high middle and low peripheral urban area. The spatial distribution pattern of population reachable locational entropy changes more obviously, with the high-value area of locational entropy concentrated from the central part in the walking case to sporadic scattering during cycling, and then to the high-value area of driving concentrated in the eastern part of the study area. The locational entropy values of the sub-districts are increasing and decreasing. Combined with the effective service range of UPGS under different travel modes, it shows that a longer travel distance will increase the overall per capita level of UPGS acquisition in the study area. Although each sub-district unit has relatively more services, it does not mean that the level of the sub-district is higher than the overall level, and the spatial distribution maps of locational entropy also reflect the existence of human green imbalance in the main urban area of Fuzhou.

With the changing of travel modes, the service proportion of district-level parks and special parks has increased significantly, while the proportion of community parks remains low, and the proportion of municipal parks is absolutely superior. The overall accessibility and per capita accessibility of sub-districts show similar spatial distribution patterns under different travel modes, which are "high around and low in the middle" and "centered on the Minjiang River and dispersed to the north and south, respectively". The Gini coefficients of building accessibility and per capita accessibility decreased gradually with the upgrading of transportation modes, and the degree of variation in park accessibility decreased and spatial equity increased.

### 4.3. Specific application of Fuzhou urban planning based on spatial equity evaluation

This evaluation system can be used as a decision-making tool to provide appropriate guidance to policy makers such as governments, urban planners, and facility operators, with equity as a fundamental objective [42]. According to the results of location entropy calculation, there is spatial variation in the distribution of UPGS resources among sub-districts, and the "human-green space matching" relationship is poor in some units, i.e., there is spatial inequity in the distribution of UPGS in the main urban area of Fuzhou. However, according to the results of the Gini coefficient, the spatial variability of UPGS service supply is small and the resource distribution is reasonable, the accessibility of UPGS between building units is significantly different, i.e., the spatial inequity is significant. The overall accessibility of UPGS between sub-districts is different, but it shows a strong positive correlation with the distribution of sub-district population, indicating that the UPGS layout matches the population demand and the resource distribution is relatively reasonable [25]. The differences in per capita accessibility are relatively small and at a more equitable level. In summary, the spatial distribution of UPGS in the main urban area of Fuzhou is relatively equal.

We focus on areas where UPGS services are below the average level according to the calculation results, explore the causes of inequity, and propose improvement measures based on actual field research. According to the effective service range distribution of UPGS, the inability to enjoy UPGS services at the periphery of the study area is caused by incomplete construction and development, the lack of road networks to facilitate access to the UPGS, and other reasons. It should intensify efforts in the construction, prioritize the completion of the construction of public infrastructures, and reasonably build roads to facilitate the linkage between the residential areas and the UPGS. From the viewpoint of the per capita accessibility of parkland, city-level parks are better constructed and have an absolute advantage in providing parkland services, while district parks, community parks, and special parks account for a very small percentage. Owing to the high density of buildings in the main urban area of Fuzhou, there is little unused space in large areas, and the "community living circle" is developing, so priority should be given to increasing the number of community parks. Considering the unique functional specificity and population targeting of special parks, the government should consider the status quo and urban construction goals in an integrated manner and make comprehensive planning. Considering the entropy calculation results of the two dimensions, the inequity of UPGS in the Dongjie, Shanghai, Ninghua, Yizhou, and Cangxia sub-districts is more serious, which is caused by the high population density of these sub-districts and the large number of old neighborhoods. They need to make full use of the abandoned space for micro-renewal and micro-renovation, and give priority to building community parks to meet residents’ requirements. The supply of UPGS services and the matching of supply and demand in the Luozhou and Chengmen sub-districts are not satisfactory. It is necessary for government departments to optimize the service benefits of UPGS by improving UPGS and road systems, increasing construction investment, and standardize the management of completed parks combine with urban development planning.

### 4.4. Limitations and future directions

This study explored walking, cycling and driving as the three most preferred modes of traveling within the study area, but public transportation trips still exist. Due to the existence of the phenomenon of mixing multiple modes of travel, the calculation of public transportation trips is more difficult and future research could refine it in terms of travel patterns. There are newly built but unused residential in the city. If the vacant residential areas can be located and eliminated, the results will be further accurate. In this study, although the grading of parklands is judged differently, the actual situation of existing parks at the same level also affects the willingness of pedestrians to travel, and it would greatly increase the authenticity of the study if the evaluation of park quality, such as aesthetics, the richness of facilities, and park safety, could be added to the evaluation of park grading. The evaluation of park quality has been touched upon in some studies [25,43,44], but a unified evaluation system has not yet been formed, and the rationality of the evaluation criteria is open to question. The evaluation is highly subjective, and the participation of all people and the development of a scientifically based park evaluation scale together with experts, scholars, and government departments will promote the progress of environmental landscape and urban planning research. This scale can also be used as a basis for urban landscape improvement, which will be beneficial for enhancing people’s happiness and building a livable city.

The connotation of equity explored in this study has three levels: whether people can reach the park green space within a certain travel range, the degree of spatial matching of supply and demand in each Sub-district, and the variability of benefits among spatial units. The demand side should be considered as an undifferentiated population, but the true sense of equity in the new era should meet the diverse needs of different people and tilt the allocation of public facilities to disadvantaged groups [45]. How to define the attributes of different populations, the criteria for judging the needs of populations, and the establishment of a human centered fairness evaluation system should be the key research topics in the future.

## 5. Conclusions

This study establishes a new set of spatial equity evaluation frameworks and explores the results of spatial equity of park green spaces in Fuzhou City using a data analysis and visualization platform. The results show that: (1) The overall accessibility and per capita accessibility show similar spatial distribution patterns under different travel modes, which are "high around and low in the middle" and "centered on the Minjiang River and dispersed to the north and south, respectively" in study area. (2) The supply of UPGS services in Fuzhou is relatively adequate and at a relatively equity level, but there are some differences in the allocation of UPGS services among sub-districts. Trip modes have significant influence on UPGS spatial equity, which varies greatly among different travel modes. (3) The sub-districts on the periphery of the study area are poorly developed, which should be subject to increased UPGS and improvements to the road network and public infrastructure. Sub-district with high population density and numerous old neighborhoods should implement micro-renewal and build community parks on sight. By making planning decisions based on a comprehensive analysis and assessment of the two dimensions of service provision and supply-demand matching, urban park construction will be more precise and scientific. Our study presents a new idea for spatial equity research.

## Supporting information

S1 File Appendices. Location Entropy Under Different Transportation Modes.
(DOCX)

S2 File (RAR)
