# Supplementary material for: From service capacity to spatial equity: Accurately and comprehensively evaluating urban park green space distribution under multi-trips mode
Source: PLoS One. 2024 Jan 25;19(1):e0296629. doi: 10.1371/journal.pone.0296629 (PMC10810483; doi:10.1371/journal.pone.0296629)
Supplement: S1 File — Location Entropy Under Different Transportation Modes. (DOCX) [file pone.0296629.s001.docx]

**S1 File.Appendices.Location Entropy Under Different Transportation Modes**

| Transport modes | Location entropy | Number of sub-districts | Proportion | Name of sub-districts/sub-districts | | | | | | | |
| --- | --- | --- | --- | --- | --- | --- | --- | --- | --- | --- | --- |
| Walking | >1 | 21 | 53.8% | AnTai sub-district | | AoFeng sub-district | CangQian sub-district | ChaYuan sub-district | DongSheng sub-district | |  |
|  |  |  |  | DuiHu sub-district | | GuShan sub-district | GuXi sub-district | HongShan sub-district | HouZhou sub-district | |  |
|  |  |  |  | HuaDa sub-district | | JianXin sub-district | LinJiang sub-district | NanJie sub-district | SanChajie sub-district | |  |
|  |  |  |  | ShangDu sub-district | | XiaDu sub-district | XinGang sub-district | YangZhong sub-district | YangZhou sub-district | |  |
|  |  |  |  | YueFeng sub-district | |  |  |  |  | |  |
|  | 0-1 | 18 | 46.2% | CangShan sub-district | | CangXia sub-district | ChaTing sub-district | ChengMen sub-district | DongJie sub-district | |  |
|  |  |  |  | GaiShan sub-district | | GuDong sub-district | JinShan sub-district | LuoZhou sub-district | NingHua sub-district | |  |
|  |  |  |  | ShangHai sub-district | | ShuiBu sub-district | WangZhuang sub-district | WenQuan sub-district | WuFeng sub-district | |  |
|  |  |  |  | XiangYuan sub-district | | XinDian sub-district | YiZhou sub-district |  |  | |  |
| Cycling | >1 | 21 | 53.8% | AnTai sub-district | | AoFeng sub-district | CangQian sub-district | ChaYuan sub-district | DongSheng sub-district | |  |
|  |  |  |  | GuShan sub-district | | GuXi sub-district | HongShan sub-district | HouZhou sub-district | HuaDa sub-district | |  |
|  |  |  |  | JianXin sub-district | | JinShan sub-district | LinJiang sub-district | SanChajie sub-district | WangZhuang sub-district | |  |
|  |  |  |  | XiaDu sub-district | | XiangYuan sub-district | XinDian sub-district | XinGang sub-district | YangZhong sub-district | |  |
|  |  |  |  | YangZhou sub-district |  | |  |  |  | |  |
|  | 0-1 | 18 | 46.2% | CangShan sub-district | | CangXia sub-district | ChaTing sub-district | ChengMen sub-district | DongJie sub-district | |  |
|  |  |  |  | DuiHu sub-district | | GaiShan sub-district | GuDong sub-district | LuoZhou sub-district | NanJie sub-district | |  |
|  |  |  |  | NingHua sub-district | | ShangDu sub-district | ShangHai sub-district | ShuiBu sub-district | WenQuan sub-district | |  |
|  |  |  |  | WuFeng sub-district | | YiZhou sub-district | YueFeng sub-district |  |  | |  |
| Driving | >1 | 19 | 48.7% | AnTai sub-district | | AoFeng sub-district | CangQian sub-district | ChaYuan sub-district | DongSheng sub-district | |  |
|  |  |  |  | GuShan sub-district | | GuXi sub-district | HouZhou sub-district | HuaDa sub-district | JianXin sub-district | |  |
|  |  |  |  | JinShan sub-district | | LinJiang sub-district | SanChajie sub-district | XiaDu sub-district | XiangYuan sub-district | |  |
|  |  |  |  | XinDian sub-district | | XinGang sub-district | YangZhong sub-district | YangZhou sub-district | |  |  |
|  | 0-1 | 20 | 51.3% | CangShan sub-district | | CangXia sub-district | ChaTing sub-district | ChengMen sub-district | DongJie sub-district | |  |
|  |  |  |  | DuiHu sub-district | | GaiShan sub-district | GuDong sub-district | HongShan sub-district | LuoZhou sub-district | |  |
|  |  |  |  | NanJie sub-district | | NingHua sub-district | ShangDu sub-district | ShangHai sub-district | ShuiBu sub-district | |  |
|  |  |  |  | WangZhuang sub-district | | WenQuan sub-district | WuFeng sub-district | YiZhou sub-district | YueFeng sub-district | |  |
